# The paradoxical relationship between emotion regulation and gambling-related cognitive biases

**DOI:** 10.1371/journal.pone.0220668

**Published:** 2019-08-05

**Authors:** Cristian M. Ruiz de Lara, Juan F. Navas, José C. Perales

**Affiliations:** Mind, Brain and Behavior Research Center (CIMCYC), University of Granada, Granada, Spain; University of Auckland, NEW ZEALAND

## Abstract

**Background:**

Gambling behavior presents substantial individual variability regarding its severity, manifestations, and psychological correlates. Specifically, differences in emotion regulation, impulsivity, and cognitive distortions have been identified as crucial to describe individual profiles with implications for the prevention, prognosis, and treatment of gambling disorder (GD).

**Aims and method:**

The aim of the present study was to investigate the associations of gambling-related cognitions (measured according to the GRCS model) with impulsivity (UPPS-P model) and emotion regulation (CERQ model), in a sample of 246 gamblers with different levels of gambling involvement, using mixed-effects modelling to isolate theoretically relevant associations while controlling for the potentially confounding effects of sociodemographic and clinical covariates.

**Results:**

Affective/motivational dimensions of UPPS-P impulsivity *positive urgency* and *sensation seeking*, on the one hand, and CERQ emotion regulation strategies *reappraisal*, *rumination* and *blaming others*, on the other, independently and significantly predicted distorted gambling-related cognitions.

**Conclusions:**

These results (a) reinforce the ones of previous studies stressing the relevance of emotional and motivational processes in the emergence of gambling-related cognitive distortions; and (b) replicate the seemingly paradoxical finding that gamblers use emotion regulation strategies customarily considered as adaptive (i.e. reappraisal) to strengthen and justify their biased beliefs about gambling outcomes and controllability.

## Introduction

Gambling disorder (GD) is a behavioral addiction [1] characterized by preoccupation and loss of control over gambling behavior, and persistent gambling engagement despite adverse consequences [2], with a worldwide estimated lifetime prevalence ranging between 0.7 and 6.5% [3]. GD is associated with a wide repertoire of negative consequences [4], and is also frequently comorbid with mood and anxiety disorders [5,6], substance-use disorders [7,8], and general health problems [9].

Over the last years, there has been a significant increase in GD research, and important advances have been made at elucidating its etiology and vulnerability markers [10,11]. Accordingly, GD must be regarded as a multifaceted phenomenon [12], influenced by a variety of risk factors, including genetic dispositions [13,14], sociodemographic and exposure variables [15,16], personality factors [17,18], family antecedents of GD or substance-use disorders [19,20], and adverse events during childhood [21,22].

With regard to more proximal causes, converging evidence shows the relevance of a number of individual processes and predispositions regarding gambling course and development [23,24]. Specifically, a large body of research has identified emotion regulation deficits [12,25,26], impulsivity [17,27,28], and gambling-related cognitive distortions [29,30], among the most critical variables contributing to GD.

More specifically, and in direct relation with the aims of the present study, emotion regulation refers to conscious and unconscious actions, either overt or covert, involved in monitoring, evaluating, and modulating emotional reactions [31,32], and converging evidence emphasizes its central role in GD [12,33]. On the one hand, individuals with GD (IGD) tend to use gambling itself as an emotion regulation strategy [34,35]. The successful attenuation of negative emotions through gambling engagement can operate as a source of negative reinforcement, predisposing individuals to maintain gambling [36,37]. Accordingly, studies have found that the use of gambling to cope with negative emotions is associated with worse gambling outcomes, higher severity, and the number of gambling activities practiced [34,37,38]. On the other hand, IGD also present anomalies in covert emotion regulation, namely conscious or unconscious mental processes used to attenuate negative emotions or enhance positive ones [25,39,40]. Specifically, IGD are more prone to use maladaptive emotion regulation strategies, such as emotional suppression [41,42], and less prone to use adaptive ones, as reappraisal [26].

Relatedly, GD is associated with impulsivity, and particularly with its emotional aspects [17,43,44]. Available evidence also shows that emotion-driven impulsivity and emotion dysregulation are tightly linked [45–48], and that problem gambling can be motivated both by the impulsive desire to avoid negative mood states and by the impulsive desire to maintain and enhance positive mood states [49].

### Theoretical models of the role of emotion regulation in problematic gambling

Current etiological models attribute a key role to emotion regulation in the vulnerability, course, and prognosis of GD. In the seminal Pathways Model [12], *conditioned gamblers* are those whose gambling has become problematic as a consequence of the reinforcement schedules and other contingencies present in gambling setting and devices, but do not present further complications. The *emotionally vulnerable* gambler subtype is however described as more prone to suffer from depression and anxiety, and also to use gambling as a strategy to cope with negative affect, whereas the *impulsivist/antisocial* subtype presents more impulsivity and a heightened risk of comorbid externalizing problems. Extensive evidence shows that comorbidity between addictions and other externalizing problems is driven by a common transdiagnostic factor that largely overlaps with negative urgency, and has been described as a form of emotion dysregulation [50, 51].

The recently proposed Gambling Space Model (GSM, [52]) reformulates the Pathways Model from a dimensional perspective. The model proposes the existence of four dimensions that would be relevant for the characterization of risky gambling and GD. The first two of them comprise the gambler’s sensitivity to the positively and negatively reinforcing properties of gambling (with emotionally vulnerable gamblers scoring high in their sensitivity to negatively reinforcing gambling properties, namely using gambling to cope). The third one, general emotion dysregulation, mostly coincides with the tendency to lose control in negative emotional circumstances, so gamblers in the high end of this dimension would largely overlap with impulsivist/antisocial ones. Finally, the fourth dimension, self-deceptive reasoning, captures the tendency to use elaborated reasoning strategies to justify heavy gambling and disguise its negative consequences. This fourth dimension allows the characterization of a new phenotype, sociodemographically characterized by younger age and higher education, and psychologically characterized by particularly strong gambling-related cognitive distortions, and heightened sensitivity to the rewarding features of gambling activities [53,54]. This subtype is becoming progressively more prevalent [55–57], and seems difficult to accommodate into the Pathways Model, but would be easily described in the GSM as the combination of high scores in the dimensions for self-deceptive reasoning and the sensitivity to gambling rewarding properties.

### The interplay between emotion regulation and gambling cognitions

Gambling-related cognitions are among the most reliable indices of risky/disordered gambling, and some of them can be defined as cognitive biases regargding one’s ability to predict and influence gambling outcomes [58,59]. Nevertheless, despite being defined as cognitions, these beliefs have been consistently linked to non-strictly-cognitive constructs. According to Michalczuk and colleagues [44], for example, impulsivity in IGD is associated with gambling biases because impulsive behavior in decision making contexts can predispose gamblers to accept distorted beliefs without questioning. However, this interpretation fails to account for the finding that cognitive biases correlate more robustly and systematically with emotional and motivational aspects of impulsivity (sensation seeking, positive urgency and negative urgency) than with its purely cognitive facets (lack of perseverance and premeditation) [60].

Alternatively, the GSM conceptualizes distorted gambling cognitions as a manifestation of self-deceptive reasoning, namely the proneness to distort reality in a self-serving way, and generates two new predictions. First, as far as gambling cognitions are motivated, emotional and motivational dimensions of impulsivity (positive and negative urgency, and sensation seeking) are expected to be more strongly connected to them than purely cognitive facets (lack of perseverance, and lack of premeditation). This prediction arises from the assumption that cognitive biases are fueled by the same emotions and motives that trigger affect-driven impulsivity.

And second, the GSM hypothesizes a substantial overlap between biased gambling-related cognitions and elaborated emotion regulation strategies. In other words, it counterintuitively predicts that putatively adaptive emotion regulation strategies used by healthy individuals to deal with negative emotions (e.g. different forms of reappraisal, re-attribution, or refocusing, generally associated with positive outcomes) can be used by IGD and risky gamblers to deal with negative events (e.g. losses) and enhance positive emotions that help them justify their excessive gambling. In line with this prediction, two recent studies by Navas and colleagues [25], and Jara-Rizzo et al. [61] have shown that treatment-seeking IGD and community gamblers with stronger cognitive distortions are more prone to use putatively adaptive emotion regulation strategies (i.e. putting into perspective, from the Cognitive Emotion Regulation Questionnaire, CERQ [62], and reappraisal form the Emotion Regulation Questionnaire, ERQ [63] than healthy controls. In other words, elaborated emotion regulation strategies, including those customarily regarded as adaptive, can contribute to cognitive distortions and gambling maintenance.

### Study aims

The present study is aimed at corroborating the two abovementioned predictions regarding the relationship between emotion regulation and gambling related cognitions. First, the closer relationship of gambling cognitions with emotional/motivational aspects of impulsivity than with its cognitive components. And second, the (seemingly counterintuitive) direct relationship between gambling cognitions and emotion regulation strategies that could reflect gamblers’ attempts to distort reality in a self-serving way.

The present study thus attempts a conceptual replication of the pattern of results reported by Navas et al. [25], and Jara-Rizzo et al. [61], specifically regarding the relationships between emotion regulation and gambling cognitions. Beyond the face value of conceptual replications, in the present study we used an emotion regulation questionnaire (CERQ) assessing a collection of strategies that allows to identify those that can be potentially used for self-deception (e.g. different types of reappraisal or blaming others). Although this is the same instrument used in Navas et al. [25], here we use a much larger sample, and the methodology is improved in a number of ways. Additionally, the existence of previous results allows a research strategy that is more confirmatory than exploratory (and thus restricts the number of models to consider).

The hypotheses were tested in a heterogeneous sample of recreational gamblers and IGD from Spanish communities. As output variables, gambling severity was measured using the South Oaks Gambling Screen (SOGS, Spanish version [64]), and gambling-related cognitive distortions were assessed with the Gambling Related Cognitions Scale (GRCS [65]). Relevant predictors were impulsivity dimensions included in the UPPS-P model (negative urgency, positive urgency, sensation seeking, lack of premeditation, and lack of perseverance [66]), and dispositional use of emotion regulation strategies included in the CERQ [62], both dysfunctional (i.e. catastrophizing, rumination, blaming oneself, and blaming others) and putatively adaptive or functional (i.e. positive refocusing, refocusing on planning, positive reappraisal, acceptance, and putting in perspective). In line with the premises outlined above, we expect (a) emotional and motivational dimensions of impulsivity (urgencies and sensation seeking), to be more strongly associated with cognitive distortions than cognitive impulsivity (lack of perseverance and premeditation); and (b) dispositional use of ego-protecting cognitive strategies of emotion regulation (particularly putting into perspective and reappraisal, according to previous studies) to be positively associated with gambling-related cognitive distortions.

## Methods

### Participants and procedure

The study sample comprised 246 gamblers, including 30 treatment-seeking patients with DSM-5-based GD diagnosis, 20 community gamblers who potentially met GD criteria (as assessed by SOGS) but were not in treatment, and 196 community gamblers that did not meet GD diagnostic criteria.

Patients were recruited from a behavioral addictions rehabilitation center in Granada, Spain (AGRAJER, Asociación Granadina de Jugadores de Azar en Rehabilitación). Community gamblers were initially recruited via social media and advertisements, and researchers also visited university schools and administered a brief screening battery to identify individuals who participate in gambling activities. Recruitment was intended to cover the whole range of gambling involvement, from occasional to heavy. Potential participants from any source who had gambled at least once were invited to complete the research protocol.

Inclusion criteria for the whole sample were: being at least 18 years old, speaking fluent Spanish, and life-time involvement in any gambling activity, regardless of the money wagered. Although no specific time period was established to define lifetime gambling involvement, only one participant from the whole sample reported not having gambled during the previous year.

The sociodemographic and relevant clinical information collected is depicted in Table 1 (upper panel). Sociodemographic information included age, gender, years of education, and monthly income (according to 6 categories, see Table note). Relevant clinical information included gambling severity and preferred gambling modality. The rightmost column in Table 1 shows the Bayes Factors for the comparisons, in all variables, between IGDs and recreational gamblers. BFs were computed using a Bayesian Mann-Whitney U tests (except for gender, for which a Bayesian contingency table test was performed), with the default priors and specifications in JASP statistical software. In general, BF > 3 is to be interpreted as substantially supportive of the alternative hypothesis of a difference between the groups in the corresponding variable, whereas BF < 1/3 supports the null (no difference between the groups). 1/3 < BF < 3 provides only anecdotal evidence.

**Table 1:** Descriptive data of the study.

	*Total sample* *(n = 246)*		*IGD*^*a*^ *[105]* *(n = 50)*		*Recreational gamblers* *(n = 196)*		
	*Mean (SD)*		*Mean (SD)*		*Mean (SD)*		*BF*_*10*_
**Age**	33.14 (13.88)		33.78 (11.46)		32.97 (14.47)		0.187
**Gender**	82 females		1 female		81 females		2.270 x 10^7^
**Years of education**	15.23 (3.96)		13.82 (3.91)		15.60 (3.90)		8.523
Monthly income*	4.13 (1.58)		4.22 (1.61)		4.11 (1.58)		0.161
**Gambling severity (SOGS)**	2.55 (3.95)		9.54 (3.12)		0.73 (1.00)		5.972 x 10^8^
Preferred gambling modality** ***[83]***	Type I	Type II	Type I	Type II	Type I	Type II	
	n = 77	n = 128	n = 20	n = 23	n = 57	n = 105	
**Gambling cognitions (GRCS)**^***b***^							
Predictive control	2.48 (1.51)		3.93 (1.68)		2.10 (1.22)		64413.72
Illusion of control	1.78 (1.20)		2.69 (1.57)		1.55 (0.96)		210.39
Interpretative bias	2.53 (1.73)		4.19 (1.88)		2.09 (1.40)		6615.03
Gambling expectancies	2.39 (1.51)		3.94 (1.90)		1.99 (1.08)		8803.14
Inability to stop gambling	1.78 (1.38)		3.89 (1.55)		1.24 (0.60)		64484.83
**Impulsivity (UPPS-P)**^***b***^							
Positive urgency	2.53 (0.65)		2.79 (0.59)		2.46 (0.65)		81.80
Negative urgency	2.52 (0.77)		2.94 (0.71)		2.40 (0.74)		107.37
Sensation seeking	2.39 (0.80)		2.58 (0.84)		2.34 (0.78)		0.62
Lack of premeditation	1.84 (0.61)		2.12 (0.66)		1.76 (0.58)		7.27
Lack of perseverance	1.71 (0.62)		2.01 (0.65)		1.63 (0.59)		28.35
**Emotion regulation strategies (CERQ)**^***b***^							
Putting into perspective	3.36 (1.01)		3.34 (1.03)		3.36 (1.00)		0.19
Positive refocusing	2.53 (1.06)		2.77 (1.11)		2.47 (1.04)		0.74
Positive reappraisal	3.39 (1.11)		3.31 (1.19)		3.41 (1.09)		0.18
Acceptance	3.56 (1.04)		3.96 (0.95)		3.46 (1.04)		21.62
Refocus on planning	3.76 (1.00)		3.92 (0.94)		3.72 (1.02)		0.66
Self-blame	2.52 (1.06)		3.31 (1.23)		2.31 (0.91)		640.93
Other-blame	1.91 (0.80)		2.02 (1.08)		1.88 (0.71)		0.18
Rumination	3.17 (1.05)		3.50 (1.10)		3.08 (1.01)		29.11
Catastrophizing	2.17 (0.90)		2.85 (1.01)		1.98 (0.77)		180841.02

Note:

^a^ Community gamblers with SOGS severity score ≥ 5 [105] and treatment seeking gamblers.

^***b***^ GRCS range [1–7]; UPPS-P range [1–4]; CERQ range [1–5]

* Monthly income in Euros, 1: ≤ 600; 2: 601–1000; 3: 1001–1500; 4: 1501–2000; 5: 2001–2500¸; 6 ≥ 2500.

** Preferred gambling modality was classified according to Navas et al.’s criteria [83]. Type I: Cards, casino games, skills and sports bets; Type II: Lotteries, pools, bingo, and slot machines. Missing data [Individuals with gambling disorder/Recreational gamblers]: Age = 0/7; gender = 0/5; years of education = 1/7; Socio-economic status = 0/5; Preferred gambling modality = 7/34; GRCS = 0/1; UPPS-P = 0/6; CERQ = 0/7.

Complementarily, among IGDs, 8% gambled at least once a month but less than once a week, 38% gambled at least once a week but less than once a day, and 54% gambled daily, in at least one of the games in the list. Among recreational gamblers, 1 participant (0.5%) had not gambled in the last year, 37.2% had gambled at least once in the last year, but less than once a month, 26.5% had gambled at least once a month, but less than once a week, 33.2% had gambled at least once a week, but less than once a day, and only 2.6% gambled daily, in at least one of the games in the list.

114 participants were assessed face-to-face by one of the researchers, using paper-and-pencil instruments, 92 participants were provided with assessment materials to complete at home, and 40 participants completed the questionnaires using a protocol created in LimeSurvey Pro 2.50 (LimeSurvey GmbH, Carsten Schmitz, HRB 137625).

Participants were informed about the aims and instructions, either face-to-face or by email, and were required to sign the informed consent prior to participation. Before giving permission to access the questionnaires’ platform, online participants were asked to read and understand the aims and instructions, and to give explicit consent to participate in the study. Assessment were performed by psychologists, and supervised by a researcher with seven years of experience in psychological assessment.

The assessment protocol consisted of various self-report measures, some of which are beyond the scope of the present study, and have been previously reported [34] (with an overlap of 76,42% between samples), or will be reported elsewhere. In addition, 21 of the 30 IGD in treatment were proposed to participate in a larger assessment protocol (programmed on a different session). This protocol included neuropsychological tasks and an fMRI session, and will be presented in future reports. Data were collected between October 2015 and December 2017.

The procedure was performed in accordance with the declaration of Helsinki and approved by the Ethics Committee of the University of Granada, as part of the PSI2013-45055-P and PSI2017-85488-P research projects (last author is the principal researcher).

### Instruments

The Gambling-Related Cognitions Scale (GRCS [65]) was used to assess gambling cognitions. The GRCS is based on a hierarchical model with five intercorrelated dimensions included in a higher order factor [65]. The first three cognitions are based on early research on pathological gambling-related cognitive biases [66,67], namely predictive control, illusion of control and interpretative bias. The other two cognitions are not strictly considered biases, but pervasive beliefs, adopted from substance-use disorders research [68], and include gambling expectancies and inability to stop gambling. Recent evidence shows that GRCS score is a robust gambling disorder predictor [69,70], and accounts for a significant amount of gambling disorder variance [30,71,72].

We used UPPS-P questionnaire [73] to assess impulsivity. According to this model, impulsivity comprises five dimensions: positive urgency, negative urgency, lack of premeditation, lack of perseverance and sensation seeking [73]. This model has been widely used in GD research with promising results [74]. A large body of research confirms significantly higher impulsivity scores in IGD, compared to controls [44,47,75]. Moreover, this approach has been included in recent theoretical models of GD [52] in an attempt to characterize different GD profiles.

Emotion regulation strategies were assessed using the Cognitive Emotion Regulation Questionnaire (CERQ [62], Spanish version [76]). This tool comprises nine different strategies of emotional regulation triggered by negative valence events. These strategies have been divided into two different clusters depending on whether they contribute to emotional well-being and adaptive behaviors or, on the contrary, they are associated with distress and psychopathological disturbances. Among the former are included: (i) *putting into perspective*, (ii) *positive refocusing*, (iii) *positive reappraisal*, (iv) *acceptance*, and (v) *refocus on planning*. The later encompass: (i) *self-blame*, (ii) *other-blame*, (iii) *rumination*, and (iv) *catastrophizing*.

The South Oaks Gambling Screen (SOGS [77], Spanish version [64]) was used to evaluate gambling severity. This is a 20-item self-report questionnaire that assesses key symptoms and common gambling-related problems. The total score ranges from 0 to 20, and can be used to determine gambling clinical status. Scores between 0 and 2 correspond to non-problem gamblers, scores between 3 and 4 are indicative of risky or problematic gambling and scores between 5 and 20 define the participant as probable pathological gambler [77]. The Spanish version of the questionnaire has shown adequate reliability and validity in general population as well as in pathological gamblers (test-retest reliability, 0.98; internal consistency, 0.94; and convergent validity, 0.92 [64]). In general, correlation between SOGS scores, DSM diagnostic criteria and gambling frequency and severity indices range from moderate to high [78].

### Statistical analysis

In order to investigate the associations between input and output variables involved in central hypotheses, hierarchical linear mixed-effects (LME) modelling, as implemented in the *nlme* R package (R Core Team, 2018 [79]) was used. Mixed-models methodology is preferable over simple regression for its less restrictive data requirements, higher flexibility, and capacity to handle missing data [80].

Given that sample size was based on availability, no a priori power analysis was feasible. However, given the large number of observations per relevant construct, the large sample size, and the limited number of predictors per model, statistical power is not expected to be a problem.

An initial model was built with participant as a random effect, and SOGS severity was included by default as fixed effect (this was done to verify that associations between input and output variables are not exclusively accounted for gambling severity). The different subscales of the GRCS questionnaire (output variables) were considered as levels of a fixed within-participant factor, and the SOGS x GRCS subscale interaction was also included in the model. Covariates (age, monthly income, education years, and gender) were included in the initial model but remained for further analyses only if they yielded significant effects (as tested using a t-test for the corresponding effect, with a relatively lenient p ≤ 0.10), and the same was done with covariate x GRCS subscale interactions. To facilitate the interpretation of effect estimates, and avoid convergence problems, all continuous variables were scaled and zero-centered prior to analyses. The final H0 model thus contained participant in the random part, and SOGS severity, GRCS subscale, SOGS x GRCS subscale, and all the covariates and their interactions with GRCS subscale with significant (p< = 0.10) contributions to the initial model (please note that the lenient threshold is used only for covariate inclusion in the model, that is, to make sure no relevant covariates are left out).

A first H1 model tested the associations between impulsivity dimensions and gambling-related cognitions. Upon the H0 model, each UPPS-P dimension was included if (a) its inclusion contributed to model fit (forward test), and (b) its exclusion from a saturated model with all UPPS-P dimensions substantially hampered model fit (backward test). After considering marginal effects of UPPS-P dimensions, the same procedure was followed by UPPS-P dimension x GRCS subscale interactions (i.e. differential effects of UPPS-P dimensions for each of the cognitions in the GRCS). Substantial UPPS-P dimension x GRCS subscale interactions were followed with GRCS subscale by subscale regressions. Model fit decisions were made on the basis of two criteria: the Akaike Information Criterion (AIC [81]) and the Likelihood-Ratio test. A second H1 model was built, using the same procedure, to test associations of CERQ emotion regulation scores with GRCS cognitions. This procedure ensures robustness of predictor effects across the presence and absence of other potential predictors.

## Results

Descriptive data for GRCS, UPPS-P and CERQ are shown in Table 1 (lower panel).

A first model was built with GRCS scores (in the five GRCS subscales) as the output variable, participant as random-effects factor, and age, gender, education years, monthly income, SOGS, and GRCS subscale as fixed-effects factors. [Please note that each participant had 5 GRCS scores (1 per GRCS subscale), but GRCS score was treated as a single dependent variable, with GRCS subscale treated as a within-participant factor]. Additionally, age, gender, education years, monthly income, and SOGS interactions with GRCS subscale (representing potentially differential effects of covariates across different GRCS cognitions) also entered the model as fixed-effects factors. Fitting was performed with the restricted maximum likelihood (REML) estimation approach. Running this model yielded significant (p < 0.10; see statistical analyses for a justification of this threshold) effects for age (t = -4.05, p < 0.001), education years (t = -1.72, p = 0.087), income (t = -1.93, p = 0.055), and SOGS (t = 9.046, p < 0.001). GRCS subscale interacted with age (maximum t = 5.07, minimum p < 0.001, across interaction contrasts), and SOGS (maximum t = 4.82, minimum p < 0.001). In other words, the final H0 model included age, education, income, SOGS, GRCS subscale, age x GRCS subscale, and SOGS x GRCS subscale in the fixed part, and participant in the random part. This model was used for further comparisons involving theoretically relevant factors.

**Fig 1:**
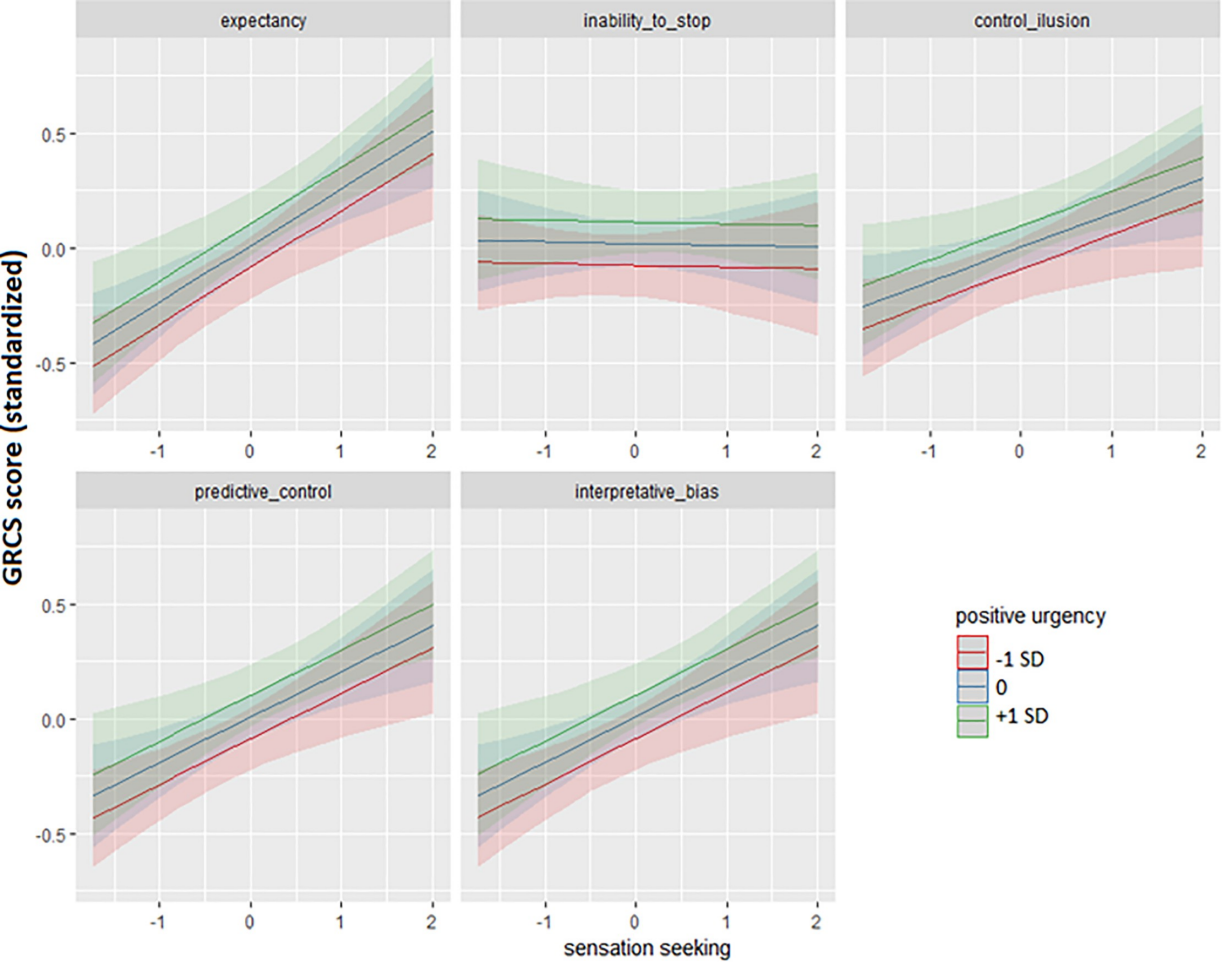
Associations of UPPS-P positive urgency and sensation seeking with scores across GRCS subscales (as predicted by the best-fitting UPPS-P + covariates model).

When UPPS-P scores in its different dimensions were used as predictors (upon the H0 model), only positive urgency and sensation seeking passed the forward and backward tests (ΔAIC = -10.768, L.Ratio = 10.768, p < 0.001; ΔAIC = -17.185, L.Ratio = 19.185, p < 0.001; ΔAIC = - 2.418, L.Ratio = 4.418 p = 0.036; and ΔAIC = -8.215, L.Ratio = 10.215, p = 0.001, for the positive urgency and sensation seeking forward tests, and the corresponding backward tests, respectively). Among UPPS x GRCS subscale interactions, only the sensation seeking x GRCS subscale interaction passed both the backward and forward tests [ΔAIC = - 22.306, L.Ratio = 30.306, p < 0.001; and ΔAIC = - 17.434, L.Ratio = 25.435, p < 0.001; all comparisons were performed fitting models with with the maximum likelihood (ML) estimation approach]. In other words, the best-fitting model included the same effects as the H0 model, plus positive urgency, sensation-seeking, and the sensation seeking x GRCS subscale interaction. Predicted GRCS values from the best-fitting model are depicted in Fig 1. The five panels in the Figure represent the effects of positive urgency (different lines), and sensation seeking (horizontal axis), for the five GRCS subscales (gambling expectancy, inability to stop gambling, control illusion, predictive control, and interpretative bias), respectively.

The effects of positive urgency and the sensation seeking x GRCS subscale interaction were followed by regression analyses for each GRCS subscale separately (using positive urgency and sensation seeking as main predictors, and age, education years, income, and SOGS scores as potential confounders). These analyses yielded significant effects of positive urgency on control illusion [B = 0.147, SE = 0.067, t = 2.210, p = 0.028, R^2^_nsj_ = 0.022, CI (0.001; 0.074)], and predictive control [B = 0.146, SE = 0.058, t = 2.469, p = 0.014, R^2^_nsj_ = 0.027, CI (0.001; 0.083)]. Sensation seeking significantly influenced predictive control [B = 0.173, SE = 0.060, t = 2.908, p = 0.004, R^2^_nsj_ = 0.037, CI (0.004; 0.099)], interpretative bias [B = 0.210576, SE = 0.061, t = 3.452, p < 0.001, R^2^_nsj_ = 0.051, CI (0.010; 0.12)], and gambling expectancies [B = 0.266, SE = 0.060, t = 4.457, p < 0.001, R^2^_nsj_ = 0.082, CI (0.027; 0.16)].

**Fig 2:**
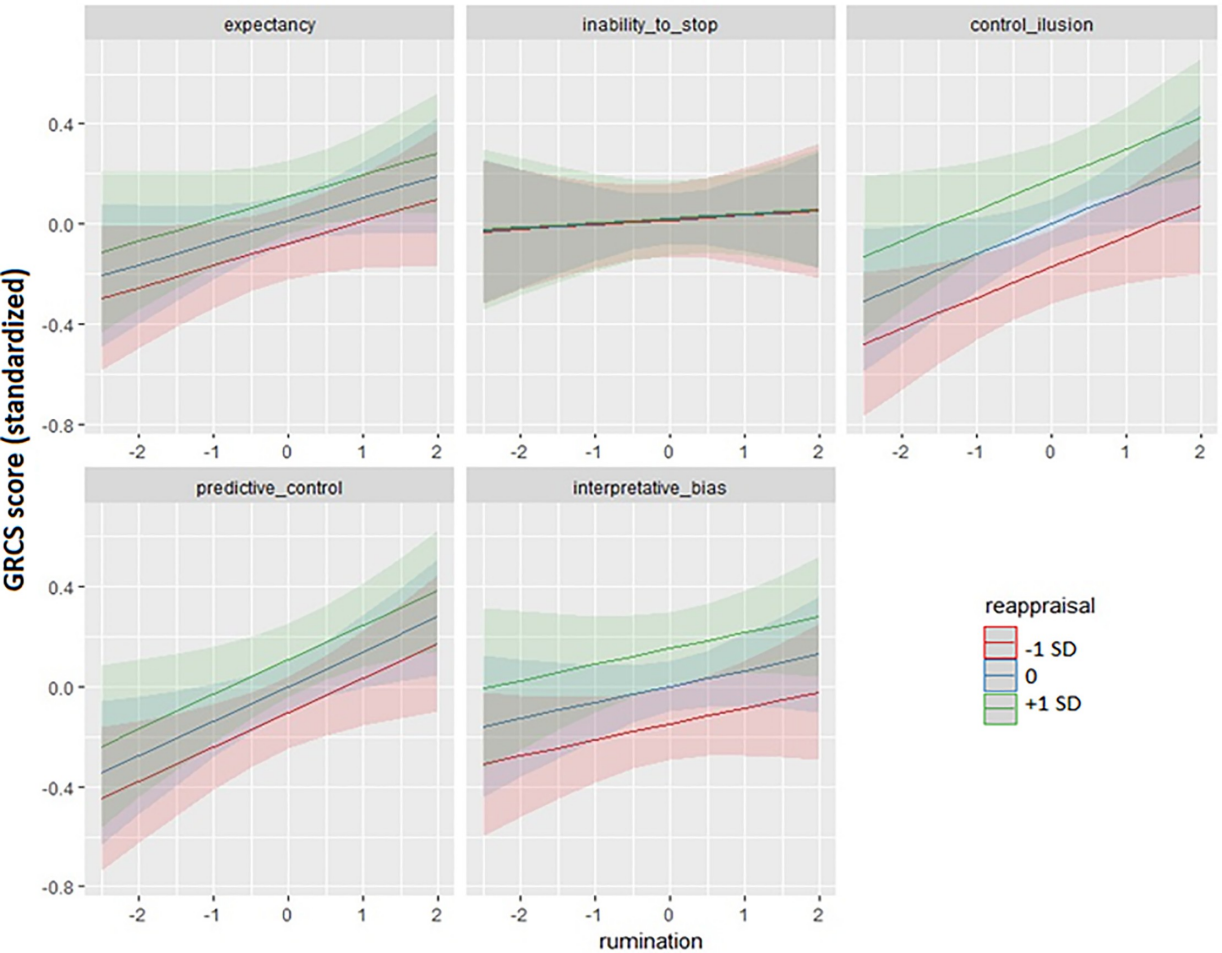
Associations of CERQ reappraisal and rumination with scores across GRCS subscales (as predicted by the best CERQ + covariates model). Note: The effect of blaming others was not found to interact with GRCS subscale, and is not shown in the figure. Effect sizes of the associations between blaming others and GRCS scores are reported in the text.

An identical analysis rationale was followed to estimate the relationships between CERQ emotion regulation strategies and GRCS cognitions. Against the H0 Model, only the strategies reappraisal and blaming others passed both the forward and the backward tests (ΔAIC = -4.616, L.Ratio = 6.616, p = 0.010; ΔAIC = -10.624, L.Ratio = 12.624, p < 0.001; ΔAIC = - 7.500, L.Ratio = 9.500 p = 0.002; and ΔAIC = -9.349, L.Ratio = 11.349, p < 0.001). Additionally, among CERQ scores x GRCS subscale interactions, both the reappraisal x GRCS subscale, and the rumination x GRCS subscale passed the forward and backward tests (ΔAIC = -8.571, L.Ratio = 16.571, p = 0.002; ΔAIC = -2.243, L.Ratio = 10.243, p < 0.037; ΔAIC = - 4.300, L.Ratio = 12.300 p = 0.015; and ΔAIC = -2.113, L.Ratio = 10.113, p = 0.039; see Fig 2). The five panels in the Figure represent the effects of reappraisal (different lines), and rumination (horizontal axis), for the five GRCS subscales (gambling expectancy, inability to stop gambling, control illusion, predictive control, and interpretative bias), respectively.

Similarly to impulsivity measures, associations between CERQ dimensions and GRCS cognitions were followed up using GRCS measure-by-measure regression analyses. In all of them, reappraisal, blaming others, and rumination scores were used as predictors, along with age, education years, income, and SOGS scores. Reappraisal use significantly predicted the strength of control illusion [B = 0.173, SE = 0.062, t = 2.795, p = 0.006, R^2^_nsj_ = 0.034, CI (0.003; 0.095)], predictive control [B = 0.112, SE = 0.055, t = 2.032, p = 0.043, R^2^_nsj_ = 0.018, CI (~0; 0.069)], and interpretative bias [B = 0.147, SE = 0.057, t = 2.596, p = 0.010, R^2^_nsj_ = 0.030, CI (0.002; 0.088)]. In accordance with its non-interactive effect, the use of blaming others significantly predicted the strength of all GRCS cognitions: inability to stop gambling [B = 0.121, SE = 0.044, t = 2.764, p = 0.006, R^2^_nsj_ = 0.030, CI (0.003; 0.094)], control illusion [B = 0.189, SE = 0.059, t = 3.200, p = 0.002, R^2^_nsj_ = 0.044, CI (0.007; 0.110)], predictive control [B = 0.155, SE = 0.053, t = 2.994, p = 0.004, R^2^_nsj_ = 0.038, CI (0.004; 0.100)], interpretative bias [B = 0.127, SE = 0.054, t = 2.328, p = 0.021, R^2^_nsj_ = 0.024, CI (0.001; 0.079)], and gambling expectancies [B = 0.160, SE = 0.054, t = 2.969, p = 0.003, R^2^_nsj_ = 0.039, CI (0.005; 0.102)]. Finally, rumination significantly predicted predictive control [B = 0.139, SE = 0.055, t = 2.537, p = 0.012, R^2^_nsj_ = 0.028, CI (0.002; 0.086)].

## Discussion

This study investigated emotion regulation predictors of gambling-related cognitions in individuals with different levels of gambling involvement. Using mixed-effects analysis to adjust for the effects of potential confounders and gambling severity, and in accordance with previous research [44,52,60], results showed that positive urgency, and sensation seeking (from the UPPS-P impulsivity scale), and reappraisal, rumination and blaming others (from the CERQ emotion regulation questionnaire) were associated with gambling cognitions (as measured by GRCS).

The association between gambling-related cognitions and impulsivity dimensions was specific for the emotional/motivational facets of impulsivity, positive urgency and sensation seeking. Negative urgency, however, was not significantly associated with gambling cognitions. Although previous studies have reported this association [44,60], it has also been shown to vanish when impulsivity dimensions are controlled for one another, and for gambling severity (see supplementary materials in Del Prete et al. [60]). Thus, despite the documented importance of negative urgency in GD severity and complications [17,44,82], it seems to hold no independent predictive value over gambling beliefs.

More importantly, the fact that negative urgency was not independently associated with gambling cognitions (in contrast with positive urgency and sensation seeking) is congruent with recent reports that gambling-related cognitions are stronger, and cognitive biases more prevalent, in gamblers who are highly sensitive to appetitive stimuli and motives [83,84]. Although it was hypothesized that, as long as cognitive biases are affect-driven and motivated, they should be linked to affect-driven impulsivity dimensions, it has been consistently shown that negative urgency is specifically linked to complications in the form of generalized externalizing problems beyond gambling [85,86]. Our results suggest that this complication pathway (probably underlying the impulsivist/antisocial cluster from the Pathways Model [12]), is mostly independent from cognitive symptomatology. Indeed, the combination of sensitivity to appetitive motives, strong cognitive distortions, and preference for certain game modalities, seems to be characteristic of an emerging cluster of problematic gamblers, as shown by recent reports [52].

In relation to the emotion regulation strategies from the CERQ model, results are closely coincident with Jara-Rizzo et al. [61], and mostly compatible with Navas et al.´s [83] findings. In the former, an association was found between reappraisal (as measured by the ERQ questionnaire) and cognitive biases. In the latter, the association between CERQ emotion regulation and gambling-related cognitive biases was restricted to the strategy putting into perspective (the potential association between reappraisal and cognitive biases vanished when emotion regulation strategies were tested against each other). Taken together, however, results confirm our hypothesis that gamblers can display relatively sophisticated emotion regulation strategies, including putatively adaptive ones in conjunction with strong cognitive distortions. Although blaming others is certainly not an adaptive strategy, it can also be effective at reframing gambling outcomes in a way that helps the gambler to maintain gambling behavior despite its negative consequences. In other words, blaming others would help gamblers to reinterpret positive outcomes as caused by personal abilities, and negative outcomes as a result of external influence.

This pattern of results bears important theoretical and clinical implications. In general terms, findings from the present study are consistent with the *Gambling Space Model* (GSM [52]). Although the DSM-5 establishes a unidimensional classification for GD severity based on the number of diagnostic criteria met by the patient, the GSM, in accordance with recent studies [10,87], and contemporary proposals turning towards dimensionality and transdiagnosis (Research Domain Criteria, RDoC [88,89]), highlights the relevance of variables that contribute to individual differences in GD, as predictors of decisive clinically-relevant indicators. The GSM was developed as an attempt to integrate these variables, and explain their implications for the behavioral and clinical manifestations of the disorder.

The two main findings in the present study regarding the GSM are: (a) the specificity of impulsivity-cognitions associations for emotion and motivation-driven dimensions of impulsivity (and the lack of predictive value of cognitive dimensions of impulsivity), and (b) the association of self-serving emotion regulation strategies with the tendency to hold biased gambling-related beliefs. Both findings reinforce the existence of a self-deceptive cognitive style where affect and its regulation play a central role.

Nonetheless, the absence of any independent link between negative urgency and cognitive distortions requires some further detailing of the model. As noted earlier, negative emotions do not seem to be particularly intrusive in self-deceptive gamblers. It could be that these gamblers are highly effective at regulating them, or alternatively, that they are not particularly prone to experience negative emotions and moods. Whatever the case is, a new prediction emerges: the low impact of negative affect in combination with strong cognitive biases should translate into high levels of problem denial and treatment reluctance or ambivalence (see [34] and [90] for similar arguments). On the other hand, this lack of relationship between negative urgency and cognitive biases reinforces the model in its conceptualization of negative urgency as a proxy for a different complication pathway in GD, namely, the malfunctioning of automatic, model-free emotion regulation mechanisms that are hypothesized to give rise to the externalizing problems that frequently co-occur with GD and other addictions [17,91].

Beyond the GSM, and the specific hypothesis of the present study, our results also have some other implications, both within and outside the gambling arena. First, the finding that gambling-related cognitions are more tightly linked to appetitive emotions and motives than to aversive ones also resonates with previous reports that gambling craving is inversely associated with positive affect, whereas alcohol craving directly correlates with negative affect [92]. In other words, at least in some cases, gambling seems to be easily triggered by a lack of positive experiences (rather than by the presence of negative ones). In view of the central importance of craving in the very definition of addictive processes, any similarities and differences between craving elicitation across addictive disorders deserves closer attention.

And second, the evidence presented here regarding the involvement of positive urgency and sensation seeking in gambling-related cognitions, along with related work showing the clinical importance of negative urgency in GD [17,44,75], is fully consistent with previous reports that the emotional and motivational aspects of impulsivity play specific and central etiological roles in the transition from risky behaviors to GD and other addictions [49,93]. Additionally, the differential involvement of positive and negative urgency in different gambling pathways (the former more related to sensitivity to rewarding properties of gambling and fueling cognitive distortions, and the latter involved in externalizing complications of gambling) adds upon the available evidence that these two aspects of emotion-driven impulsivity are theoretically distinct and have different clinical implications [60,94,95].

Indeed, the present study also bears clinical relevance. Cognitive distortions are among the main factors underlying gambling involvement, clinical status and gambling severity [96–98]. Our results show that people who are more prone to impulsive behavior under the influence of positive emotions (scoring high in positive urgency), and more strongly motivated by novel and exciting experiences (sensation seekers) are also more prone to develop gambling-related cognitive biases. Moreover, the association between sensation seeking and gambling expectancies suggests that there is a cluster of gamblers particularly motivated by gambling-triggered arousal and thrill. A number of studies [90,99] suggest that IGD with these characteristics are, in general, less aware of their gambling problems, present a weaker motivation to quit or reduce their gambling, are more likely to drop out from therapy, and are also less compliant with treatment assignments. The chances of intervention success with CBT and cognitive restructuration techniques alone may be thus thinner in these cases, and motivational intervention becomes recommendable [100,101].

The fact that the dispositional use of elaborated emotion regulation strategies also denotes vulnerability to cognitive biases offers a solution to the apparent paradox that general cognitive skills and numerical abilities do not protect gamblers from cognitive distortions [25,102,103], which is important for GD prevention. First, gambling distortions do not seem to be primarily rooted in the lack of probability, mathematical, or reasoning skills, but in motivational factors. And second, cognitive emotion regulation strategies probably require some preservation of the same executive functions that underlie such skills [104].

Finally, although in this study we were not particularly interested in sociodemographics by themselves (but only as control variables), it is worth to mention that age also emerged as a strong predictor of the strength of gambling cognitions, with older participants holding less distorted beliefs that younger ones. Once again, evidence suggests that self-deception seems to be particularly severe in an emerging cluster of gamblers, characterized by younger age, prevalence of positive and excitement-related motives, and preference for skill-based, high-arousal games. Some recent results seem to indicate that this subtype is growing in importance, but probably underrepresented in clinical and prevalence studies.

### Limitations and strengths

The present study, using a large sample intending to cover the whole range of gambling involvement, provides novel contributions to the understanding of the complex interplay between individual traits that depict different gambling profiles. The inclusion of both community and disordered gamblers allows better generalizability of the results. We used validated and reliable measures to assess gambling traits (UPPS-P, GRCS, CERQ) and appropriate mixed-effects analysis. Linear mixed-effects models are less restrictive with regard to data requirements and allow higher flexibility in the models’ specifications [80]. The method employed for a predictor to be considered significant was stringent, to ensure the soundness of the findings. Additionally, we also evaluate the potential confounding effects of a wide range of sociodemographic and clinically-relevant variables.

Findings from the present investigation should also be considered in light of several limitations. First, the cross-sectional nature of the statistical design precludes any inference regarding causal directionality. Second, the use of self-reported questionnaires to assess the constructs included in the models, and absence of objective measures of performance, may not entirely represent the cognitive and emotional processes involved. It may also influence results due to recall bias and social desirability. Third, and in relation to the previous caveat, effects sizes are mostly small (R^2^ > 0.01), or medium (R^2^ > 0.06) but not trivial (R^2^ < 0.01), according to customary conventions. These values are fully consistent with the ones reported in related work [90]. This is partially attributable to the measurement error of the scales used, but also, as mentioned earlier, to the fact that some of them were used as proxies to the construct of interest. Further research is indeed underway to find more direct ways to measure such constructs Complementarily, small effect sizes are also attributable to the fact that, in all analyses, gambling severity was controlled for: as correlations between constructs in the current sample are strongly driven by severity, any estimates of effect sizes beyond severity are likely to be conservative. Fourth, for the sake of parsimony, we restricted the selection of variables of interest and confounders based on *a priori* hypotheses. A number of alternative models could have also been built. And fifth, the sample size was not large enough to compare between different subsets of gamblers, for instance, based on their preferred gambling modality or their motives for gambling.

### Conclusion

Overall, our results delve into the understanding of individual differences and diverse gambling profiles among IGD, and cast light on apparent paradoxes regarding the relationships between gambling-related beliefs and emotional processes. More specifically, they suggest that gambling-related cognitive biases are tightly entangled with emotional and motivational processes, and, probably, cannot be effectively treated if these processes are neglected. Future research should confirm their generalizability to different samples and addictive disorders, and consider additional factors that could further delineate specific gamblers’ profiles.
